# The Impact of Equity Financing on the Performance of Capital-Constrained Supply Chain under Consumers’ Low-Carbon Preference

**DOI:** 10.3390/ijerph18052329

**Published:** 2021-02-27

**Authors:** Xiaoli Zhang, Guoyi Xiu, Fakhar Shahzad, Caiquan Duan

**Affiliations:** 1 School of Economics and Management, Harbin University of Science and Technology, Harbin 150080, China; zhangxl@hrbfu.edu.cn; 2 Department of Accounting, Harbin Finance University, Harbin 150030, China; 3 Department of Business Administration, Ilma University, Karachi 75190, Pakistan; fshahzad51@yahoo.com; 4 College of Engineering, Northeast Agricultural University, Harbin 150030, China; duancaiquan@neau.edu.cn

**Keywords:** low-carbon preference, capital constraint, bank loan, equity financing, hybrid financing

## Abstract

The reduction in carbon emissions by industrial enterprises is an important means for promoting environmental protection and achieving sustainable development. To determine the impact of carbon emissions reduction on supply chain operation and financing decision-making, in this study we designed three financing strategies, i.e., bank loan financing, equity financing, and hybrid financing (a combination of bank loan financing and equity financing), for a manufacturer (leader) and a low-carbon supply chain composed of a capital-constrained retailer, constructed Stackelberg game models, solved the equilibrium results under each financing strategy using the reverse recursion method, and revealed the financing preference of the supply chain member companies through comparative analysis. The results showed that the increase in the consumers’ low-carbon preference and equity financing ratio have positive impacts on supply chain equilibrium, a result that is opposite that for the impact of the interest rate of bank loan financing; additionally, the abovementioned three factors jointly determine the profit of the manufacturer of the low-carbon supply chain, while the retailer’s profit is affected by the equity dividend ratio. Finally, we present the conditions for the financing preference of the manufacturer and the retailer. The findings of this study can provide references for low-carbon supply chain companies to make appropriate management decisions.

## 1. Introduction

With global warming and the increasingly excessive consumption of resources, the progress of social and economic development has been severely hindered [1]. In order to prevent further environmental deterioration, nearly 200 participants at the United Nations Framework Convention on Climate Change reached an agreement and signed the Paris Agreement, aiming to achieve social and economic sustainable development [2] through the control and reduction in carbon emissions [3,4]. Based on a market survey by Wang et al. [5] and Cong et al. [6], with the increase in consumers’ low-carbon awareness, some consumers are willing to pay higher prices for low-carbon products, and some industrial enterprises with corporate social responsibility have begun to invest in carbon emissions reduction technologies [3,7]. For example, in 2017, Siemens, while using innovative and environmentally friendly technologies, reduced carbon dioxide emissions by 570 million tons while generating 38.7 billion euros in revenue and planned to cut carbon emissions by 50% in 2020 and will further realize its goal of net zero carbon emissions by 2030 [8]. Incorporating sustainability and low-carbon concepts into supply chain operation management is a win-win strategy for manufacturers and retailers, and it can create substantial environmental and economic benefits for supply chain enterprises. Evidently, sustainable low-carbon supply chain management has become a current hot topic and it has attracted widespread attention from both industry and academia.

When discussing optimal decisions regarding low-carbon supply chain operations, consumers’ low-carbon preference is usually considered, while the financial pressure of downstream supply chain retailers is often ignored. Capital constraints are one of the perpetual challenges facing supply chain operations [9]. Like manufacturers, small retailers down the supply chain are often at risk of a shortage of funds [10], a situation that has been aggravated by the COVID-19 pandemic. Based on a survey by the National Federation of Independent Business (NFIB), over 75% of small businesses in the United States have been affected by COVID-19. There are approximately 300,000 small businesses in the NFIB database, and the NFIB conducted a survey of random businesses and found that most businesses are negotiating with banks to seek loan assistance [11]. However, high financing costs are still bottlenecks that restrict the development of small and medium-sized retailers in the supply chain, and obtaining financing has become a new problem for members of the low-carbon supply chain.

The effect of bank loan financing on business performance in the one-on-one supply chain has been investigated, and the results showed that, when choosing a bank loan financing strategy, retailers with capital restraints can neither improve the efficiency of the supply chain nor achieve perfect coordination of the supply chain [12,13,14]. A single financing strategy can no longer meet the needs of corporate financing; therefore, many companies have begun to seek other external financing options, such as equity financing. Before going public in 2014, JD.com went through seven rounds of equity financing, which helped it to quickly increase its e-commerce platform market share and ultimately create RMB 100 billion in revenue [15]. In addition, retailers that have obtained equity financing can return to the supply chain to participate in market competition with a low-cost advantage and achieve sustainable development of the supply chain [16].

Until now, the operation and financing of low-carbon supply chains have rarely been examined from the perspective of consumers’ low-carbon preference and equity financing. To broaden the research scope in this field, in this study, we focused on consumers’ low-carbon preference and supply chain operations to reduce manufacturers’ carbon emissions to investigate the impact of equity financing on the low-carbon supply chain performance. We constructed three Stackelberg game models for bank loan financing, equity financing, and hybrid financing (a combination of bank loan financing and equity financing), and then drew conclusions and management implications through comparative analyses of equilibrium results of the models. This paper mainly addresses the following questions:

1. How do the interest rate of bank loan financing, the equity financing ratio, and consumers’ low-carbon preference affect carbon emissions reduction efficiency, order quantity, and profits of supply chain enterprises?
2. How does the equity dividend ratio affect the profits of the capital-constrained retailer?
3. Which financing strategy does member companies of low-carbon supply chains prefer?

The rest of this paper is organized, as follows. In Section 2, the relevant literature is reviewed and the limitations of previous studies are analyzed; additionally, the objectives and highlights of this study are presented; in Section 3, the notation and related assumptions are defined; in Section 4, three Stackelberg game financing strategy models are constructed; in Section 5, the selection conditions for the optimal financing strategy are presented; in Section 6, numerical examples are provided through numerical simulation; and, in Section 7, conclusions are drawn, and recommendations for future studies are provided.

## 2. Reviews on Literature and Motivations

In this section, we mainly review the related literature from the following three aspects: consumers’ low-carbon preference, capital-constrained supply chain, and low-carbon supply chains financing. In addition, we will give the motivation and highlights of our paper.

### 2.1. Consumers’ Low-Carbon Preference

Consumers’ environmental awareness is an important factor affecting low-carbon supply chain operations management [17]. When consumers are more environmentally aware, they are willing to pay a premium for low-carbon products [18,19]. Vanclay et al. [20] studied consumer low-carbon behaviors through carbon labelling experiments and found that consumers prefer to buy low-carbon products. Obviously, consumers’ low-carbon preference has a positive impact on their purchasing behavior, which makes low-carbon products increasingly popular among the general public [21].

The development of a low-carbon economy has become trendy worldwide. Consumers’ low-carbon preference promotes carbon emissions reduction in the supply chain [22] and it affects supply chain operations and carbon emissions reduction decisions [18,23]. According to a study conducted by Wu et al. in a market with high consumer preferences for low-carbon products, a reasonable cost-sharing ratio enables the coordination of carbon emissions reduction in the supply chain [2]. Wang et al. showed that an increase in consumers’ low-carbon preference can prompt manufacturers to increase the level of their carbon emissions reduction efforts and, thus, increase the profit of the entire supply chain [5].

Furthermore, consumers’ purchasing decisions are influenced by their low-carbon preference, which encourages manufacturers to increase their carbon emissions reduction efforts, in turn bringing more revenue to the manufacturers. Therefore, consumers’ low-carbon preference affects supply chain operation decisions. In this study, we included a coefficient of consumers’ low-carbon preference in the demand function in order to examine the effect of consumers’ low-carbon preference on carbon emissions reduction decisions. However, none of the above studies considered the impact of capital constraints on carbon emission reduction. Next, we summarize the literature on the capital-constrained supply chain.

### 2.2. Capital-Constrained Supply Chain

Adequate funding is important in ensuring the sustainability of the supply chain, and a shortage of funds leads to a risk of supply chain disruption and even bankruptcy for small and medium-sized retailers. To address supply chain capital constraints and achieve an optimal financing strategy, various financing strategies, such as trade credits and bank loan financing, have been compared. Deng et al. [12] compared trade credit financing and bank loan financing in a supply chain that is composed of one manufacturer and multiple suppliers. Kouvelis et al. [13] designed an optimal trade credit contract and demonstrated that capital-constrained retailers prefer trade credit financing. Chen [24] concluded that, under uncertain market demands, trade credits benefit supply chain member companies and they are the only way to achieve financial equilibrium. Lu et al. [25] revealed that bank loan financing is the optimal financing strategy in the case of tax asymmetry. Jing et al. [26] studied a secondary supply chain composed of a manufacturer and a capital-constrained retailer, and found that bank loan financing is more attractive than trade credit when only one financing option is available. Jing et al. [27] further compared the advantages of bank loan financing and trade credit in improving double marginalization in the supply chain. Feng et al. [28] showed thatm when a buyer chooses bank loan financing, the buyer’s profit is proportional to the bank loan amount. Ding et al. [29] examined the optimal decisions of suppliers, manufacturers, and the entire supply chain under a single financing strategy, i.e., bank loan financing and advance payments. Yun et al. [30] studied the impact of credit guarantees on financing decisions in the context of bank loan financing and how to achieve supply chain coordination.

The above studies compare supply chain performance from the perspective of a single financing strategy. As market competition intensifies, a single financing strategy can no longer meet the needs of small and medium-sized retailers. Capital-constrained retailers have started to demand equity financing or mixed internal and external financing methods [9,16,31]. Shen et al. [9] and Zhang et al. [32] found that the use of financing schemes combining bank loan financing with trade credits in the supply chain promotes a win-win situation for participants. Yang et al. [16] constructed a supply chain model that was composed of a supplier and two capital-constrained retailers to examine the impact of equity financing on supply chain performance, and showed that the manufacturer can form a supply chain alliance with one of the retailers to avoid double marginalization; the eliminated retailer can choose equity financing to return to the supply chain to participate in competition. They also analyzed the sensitivity of the retailer’s capital structure and listed the conditions for adopting equity financing. Li et al. [31] compared three financing strategies, i.e., trade credits, bank loan financing, and hybrid financing, and found that the equity financing ratio, interest rate of bank loan financing, and consumer product preference jointly affect the profits of supply chain member companies; in the case of a moderate equity financing ratio, hybrid financing should be the first choice. The abovementioned studies analyzed the capital-constrained supply chain, but did not consider carbon emissions reduction factors.

### 2.3. Low-Carbon Supply Chains Financing

In terms of low-carbon supply chain financing, to determine the impact of carbon emissions reduction on financing decisions, increasingly more investigators are paying attention to low-carbon supply chain financing. Wu et al. [3] analyzed a green supply chain that is composed of a manufacturer and a capital-constrained retailer, and found that the manufacturer invests in carbon emissions reduction technologies and the supply chain achieved a win-win situation in terms of production and carbon emissions reduction, with a more profound effectiveness of trade credit financing. Cao et al. [33] examined the impact of carbon emissions reduction on supply chain financing and performance. Qin et al. [34] found that bank credits can alleviate overproduction and facilitate optimal carbon emissions reduction; furthermore, the impact of the green financing rate on manufacturers regarding carbon emissions reduction is not always negative, and the retailers’ cost-sharing also does not always have a positive impact on manufacturers’ carbon emissions reduction. Aljazzar et al. [35] coordinated a supply chain from the perspective of carbon emissions reduction costs and showed that trade credits can improve the environmental and operating performance of the supply chain. However, these studies only addressed bank loan financing and trade credits from the perspective of carbon emissions reduction; therefore, the financing model is too single, and the authors never attempted to address the financial constraints of supply chains from the perspective of equity financing. Until now, only a few investigators, such as Yang et al., have taken a two-level green supply chain with a manufacturer and two capital-constrained retailers into account, of which one retailer re-enters the supply chain to compete using a hybrid financing strategy (bank loan financing and equity financing) [36].

### 2.4. Motivations and Highlights

The innovation and academic contributions of this paper are as follows: first, we take into account consumers’ low-carbon preference in a capital-constrained supply chain, based on which we further examine the effects of consumers’ low-carbon preference on the equilibrium decision-making and performance of enterprises; second, we include equity financing in the Stackelberg models and separately investigate the impact of the equity financing ratio and equity dividend ratio on corporate equilibrium decision-making and performance; finally, we draw conclusions by comparing three financing strategies, providing important management guidance for small and medium-sized retailers to develop financing strategies while expanding the research content that is related to low-carbon supply chain financing.

Moreover, in this study, we have made an interesting discovery: whether equity financing is conducive to retailers’ solving funding constraint problems and to manufacturers’ reducing carbon emissions are closely related to the interest rate of bank loan financing. The choice of financing strategies for supply chain participants depends on four operational factors, i.e., consumers’ low-carbon preference, interest rate of bank loan financing, equity financing ratio, and equity dividend ratio. The comparison of the equilibrium results of three financing strategies indicated that the conditions for the financing preferences of participants in the low-carbon supply chain. The results of this paper are helpful for supply chain participants to realize perfect financing strategies.

## 3. Problem Description and Assumptions

In this paper, we first constructed Stackelberg game models for a low-carbon supply chain, which consists of one manufacturer (leader) and one capital-constrained retailer(follower), similar to the application of this model in previous studies [37,38,39,40]. Currently, the manufacturer is adopting innovative and environmentally friendly technologies to produce low-carbon products to reduce carbon emissions, the retailer is ordering low-carbon products and promoting them in the market, and consumers are becoming more environmentally aware and they prefer low-carbon products. However, most retailers are small and medium-sized enterprises that face financial constraints, especially those in the startup stage. They have no initial capital and cannot order low-carbon products. In this study, we focus on how capital-constrained retailers can generate optimal ordering through financing when manufacturers invest in carbon emissions reduction. This problem can be solved under three different financing strategies, as follows: Bank loan financing (B), Equity financing (E), and hybrid financing of bank credit and equity financing (BE). By comparing the equilibrium results of three different financing strategies, the best financing strategy is provided for the low-carbon supply chain member enterprises.We make the following assumptions, so that the model is more realistic and economic viability, and Table 1 summarizes the notations definition used in this paper.

**Assumption** **1.** *Market demand function. The actual demand of the market is sensitive to retail prices. Given consumers’ preference for low-carbon products, we assume that reducing carbon emissions will increase market demand. Therefore, we use $g_{0}\left(e\right)=\theta e$ to represent the increase in market demand that is caused by efforts to reduce carbon emissions. To make the demand function economically meaningful and without loss of generality. Assuming that market demand is a linear function of the retail price of the products (p) and the level of carbon emission reduction efforts (e), and the retailer’s order quantity is equal to the actual demand of the market. Similar to the assumptions for demand function in previous studies [3,6,36,40], the market demand function can be given as:*

(1) q=α−bp+θeα−bp>0,α,b>0.

**Assumption** **2.** *Carbon emissions reduction cost of the manufacturer. The more carbon emissions reduction efforts made by manufacturers, the greater the cost will be. While as the level of carbon emission reduction efforts increases, its impact on the growth of market demand also increases. Therefore, we use a quadratic function to define the cost of carbon emissions reduction efforts: $g\left(e\right)=\frac{1}{2}ke^{2}$, where k is a constant that describes the carbon emission cost parameter. As described in references [3,6], k is typically large enough.*

**Assumption** **3.** *Assuming that the market is a product market in a single period and that, in the same operation period, the retailer orders low-carbon products from the manufacturer with the unit wholesale price of the products (ω), the order quantity is equal to the actual demand of the market (q) and it is then sold to consumers at a retail price of (p). In addition to the manufacturer’s production costs, there are no other costs between the manufacturer and retailer. It is also assumed that the operating behaviors of low-carbon supply chain member companies follow the financing norms and that there is no breach of contract between the two parties. Thus, we have $p>\omega \left(1+r_{b}\right)>c_{m}\left(1+r_{b}\right)>0$. Similar assumptions are made in previous research [3,13].*

**Assumption** **4.** *Assume that the low-carbon supply chain member companies are risk-neutral, without information asymmetry, i.e., manufacturers, retailers, banks, and investment institutions all share information [9,13].*

## 4. Low-Carbon Supply Chain Financing Strategies

### 4.1. Bank Loan Financing Strategy

**Figure 1:**
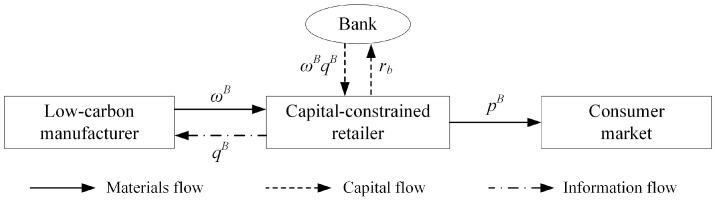
Bank loan financing strategy.

Under the bank loan financing strategy, in the initial period of operation, the retailer has no initial funds and needs to apply for loans (ωq) from a bank or other financial institutions and needs to repay the principal and interest to the bank in the amount of $\omega q(1+r_{b})$. In this section, as supported by Figure 1, the focus is on how, in a decentralized low-carbon supply chain, the manufacturer and retailer make optimal decisions to maximize profits. The following two equations describe the profit functions of the manufacturer and the retailer:

(2) $$\pi_{m}^{B}\left(\omega ,e\right)=\left(\omega -c_{m}\right)\left(\alpha -bp+\theta e\right)-\frac{1}{2}ke^{2};$$

(3) $$\pi_{r}^{B}\left(p\right)=(p-\omega \left(1+r_{b}\right)\left(\alpha -bp+\theta e\right).$$

In where, superscript *B* represents bank loan financing strategy.

This is a two-echelon Stackelberg problem and we solve it by the inverse induction method, like the research conducted by [6,39]. First, the manufacturer uses the retailer’s optimal response function to determine the wholesale price and level of carbon emissions reduction efforts; then, the retailer determines the retail price according to the manufacturer’s decision. Thus, we can get, from Equation (3), $\frac{\partial^{2}\pi_{r}^{B}\left(p\right)}{\partial p^{2}}=-2b<0$, that $\pi_{r}^{B}\left(p\right)$ is a concave function of *p*, and then set the first derivative equal to zero from Equation (3), we obtain the optimal response function of the retailer:

(4) $$p\left(\omega ,e\right)=\frac{\alpha +\theta e+b(1+r_{b})\omega }{2b}.$$

We substitute the result of Equation (4) into Equation (2), and we can get that the Hessian Matrix of $\pi_{m}^{B}\left(\omega ,e\right)$ as $H\left(\pi_{m}^{B}\right)=\left[\begin{array}{cc} -b(1+r_{b}) & \frac{\theta }{2}\\ \frac{\theta }{2} & -k \end{array}\right]$. We define $D_{1}\left(\pi_{m}^{B}\right)$, $D_{2}\left(\pi_{m}^{B}\right)$ as the first-order and second-order leading principal minors of $H(\pi_{m}^{B})$. When $D_{1}\left(\pi_{m}^{B}\right)=-b\left(1+r_{b}\right)<0$ and $D_{2}\left(\pi_{m}^{B}\right)=kb\left(1+r_{b}\right)-\frac{\theta^{2}}{4}>0$, $\pi_{m}^{B}\left(\omega ,e\right)$ is a strictly concave function of ω and *e*. Equating $\frac{\partial \pi_{m}^{B}\left(\omega ,e\right)}{\partial \omega }=0$ and $\frac{\partial \pi_{m}^{B}\left(\omega ,e\right)}{\partial e}=0$, we obtain the optimal ${\omega^{B}}^{*}$ and ${e^{B}}^{*}$:

(5) $${\omega^{B}}^{*}=c_{m}+\frac{2k(\alpha -bc_{m}\left(1+r_{b}\right))}{4kb\left(1+r_{b}\right)-\theta^{2}},$$

(6) $${e^{B}}^{*}=\frac{\theta (\alpha -bc_{m}\left(1+r_{b}\right))}{4kb\left(1+r_{b}\right)-\theta^{2}}.$$

Plugging the values of ${\omega^{B}}^{*}$ and ${e^{B}}^{*}$ into Equation (4), the optimal ${p^{B}}^{*}$ can be obtained. Subsequently, we put the values of ${p^{B}}^{*}$ and ${e^{B}}^{*}$ into Equation (1), and get the optimal ${q^{B}}^{*}$:

(7) $${p^{B}}^{*}=c_{m}\left(1+r_{b}\right)+\frac{3k\left(1+r_{b}\right)\left(\alpha -bc_{m}\left(1+r_{b}\right)\right)}{4kb\left(1+r_{b}\right)-\theta^{2}},$$

(8) $${q^{B}}^{*}=\frac{kb\left(1+r_{b}\right)\left(\alpha -bc_{m}\left(1+r_{b}\right)\right)}{4kb\left(1+r_{b}\right)-\theta^{2}}.$$

Finally, we substitute Equations (5)–(8) into Equations (2) and (3), and get the maximum profits under the bank loan financing strategy:

(9) $${\pi_{m}^{B}}^{*}=\frac{k{\left(\alpha -bc_{m}\left(1+r_{b}\right)\right)}^{2}}{2(4kb\left(1+r_{b}\right)-\theta^{2})},$$

(10) $${\pi_{r}^{B}}^{*}=\frac{k^{2}b{\left(1+r_{b}\right)}^{2}{\left(\alpha -bc_{m}\left(1+r_{b}\right)\right)}^{2}}{{\left(4kb\left(1+r_{b}\right)-\theta^{2}\right)}^{2}}.$$

**Property** **1.** *The impact of the interest rate of bank loan is given in the following results:*

$$\frac{\partial {e^{B}}^{*}}{\partial r_{b}}<0,\frac{\partial {q^{B}}^{*}}{\partial r_{b}}<0,\frac{\partial {\pi_{m}^{B}}^{*}}{\partial r_{b}}<0,\frac{\partial {\pi_{r}^{B}}^{*}}{\partial r_{b}}<0.$$

*The proof is given in Appendix A.1.*

Property 1 indicates that, as the interest rate of bank loan financing ($r_{b}$) increases, the level of the manufacturer’s carbon emissions reduction efforts (${e^{B}}^{*}$), the retailer’s order quantity (${q^{B}}^{*}$), the manufacturer’s profit (${\pi_{m}^{B}}^{*}$), and the retailer’s profit (${\pi_{r}^{B}}^{*}$) decrease accordingly. As the interest rate of bank loan financing increases, the interest the retailer needs to pay also increases, which increases the retailer’s financing costs and lowers its profit, even risking bankruptcy due to "insolvency" and, thus, weakening the retailer’s incentive to order; therefore, the manufacturer has to lower the wholesale price to prompt the retailer to increase order quantity, because the decreased market demand not only hinders the manufacture’s carbon reductions efforts, but also reduces its profit.

### 4.2. Equity Financing Strategy

**Figure 2:**
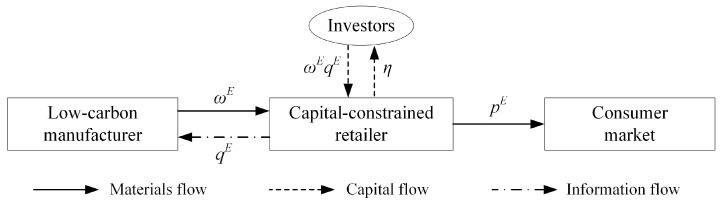
Equity financing strategy.

According to the data from Zero2IPO, in 2019, there were 8234 investment events in China’s equity market, with a total disclosed investment amount of RMB 763.094 billion [41]. Equity financing has broadened the financing channels for companies and it has become an important means for companies to explore the market [16,36]. Unlike those adopting bank loan financing, retailers opting for equity financing do not need to repay the principal and interest at the end of the period but have to pay dividends to investment institutions. Assuming that the equity dividend ratio is η, where η∈(0,1). Both the manufacturer and the retailer are pursuing a profit-maximizing business goal, as shown in Figure 2. In this section, we focus on making optimal decisions. The profit functions of the manufacturer and the retailer can be described, as follows:

(11) $$\pi_{m}^{E}\left(\omega ,e\right)=\left(\omega -c_{m}\right)\left(\alpha -bp+\theta e\right)-\frac{1}{2}ke^{2},$$

(12) $$\pi_{r}^{E}\left(p\right)=\left(1-\eta \right)\left(p-\omega \right)\left(\alpha -bp+\theta e\right).$$

In where, superscript *E* represents equity financing strategy.

First, by virtue of Equation (12), we can get that $\frac{\partial^{2}\pi_{r}^{E}\left(p\right)}{\partial p^{2}}=-2b\left(1-\eta \right)<0$, so $\pi_{r}^{E}\left(p\right)$ is a strictly concave function of *p*, and then set the first derivative equal to zero from Equation (12), we obtain the optimal response function of the retailer:

(13) $$p=\frac{\alpha +\theta e+b\omega }{2b}.$$

Next, we substitute the result of Equation (13) into Equation (11), and we can get that the Hessian Matrix of $\pi_{m}^{E}\left(\omega ,e\right)$ as $H\left(\pi_{m}^{E}\right)=\left[\begin{array}{cc} -b & \frac{\theta }{2}\\ \frac{\theta }{2} & -k \end{array}\right]$. We define $D_{1}\left(\pi_{m}^{E}\right)$, $D_{2}\left(\pi_{m}^{E}\right)$ as the first-order and second-order leading principal minors of $H(\pi_{m}^{E})$. When $D_{1}\left(\pi_{m}^{E}\right)=-b<0$ and $D_{2}\left(\pi_{m}^{E}\right)=kb-\frac{\theta^{2}}{4}>0$, $\pi_{m}^{E}\left(\omega ,e\right)$ is a strictly concave function of ω and *e*. Equating $\frac{\partial \pi_{m}^{E}\left(\omega ,e\right)}{\partial \omega }=0$ and $\frac{\partial \pi_{m}^{E}\left(\omega ,e\right)}{\partial e}=0$, we can obtain the optimal ${\omega^{E}}^{*}$, ${e^{E}}^{*}$, ${p^{E}}^{*}$ and ${q^{E}}^{*}$:

(14) $${\omega^{E}}^{*}=c_{m}+\frac{2k(\alpha -bc_{m})}{4kb-\theta^{2}},$$

(15) $${e^{E}}^{*}=\frac{\theta (\alpha -bc_{m})}{4kb-\theta^{2}},$$

(16) $${p^{E}}^{*}=c_{m}+\frac{3k(\alpha -bc_{m})}{4kb-\theta^{2}},$$

(17) $${q^{E}}^{*}=\frac{kb(\alpha -bc_{m})}{4kb-\theta^{2}}.$$

Similar to Section 2.1, we substitute Equations (13)–(16) into Equations (11) and (12), and get the maximum profits under the equity financing strategy:

(18) $${\pi_{m}^{E}}^{*}=\frac{k{\left(\alpha -bc_{m}\right)}^{2}}{2(4kb-\theta^{2})},$$

(19) $${\pi_{r}^{E}}^{*}=\frac{\left(1-\eta \right)k^{2}b{\left(\alpha -bc_{m}\right)}^{2}}{{\left(4kb-\theta^{2}\right)}^{2}}.$$

**Property** **2.** *The impact of the equity dividend ratio is given in the following results:*

$$\frac{\partial {e^{E}}^{*}}{\partial \eta }=0,\frac{\partial {q^{E}}^{*}}{\partial \eta }=0,\frac{\partial {\pi_{m}^{E}}^{*}}{\partial \eta }=0,\frac{\partial {\pi_{r}^{E}}^{*}}{\partial \eta }<0.$$

*The proof is given in Appendix A.2.*

Property 2 indicates that, under equity financing, the manufacturer’s carbon emissions reduction efforts (${e^{E}}^{*}$), the retailer’s order quantity (${q^{E}}^{*}$), and the manufacturer’s profit (${\pi_{m}^{E}}^{*}$) are all independent of the equity dividend ratio (η) and consistent with the equilibrium results without capital constraints. However, the retailer’s profit (${\pi_{r}^{E}}^{*}$) decreases as η increases.

### 4.3. Hybrid Financing Strategy

**Figure 3:**
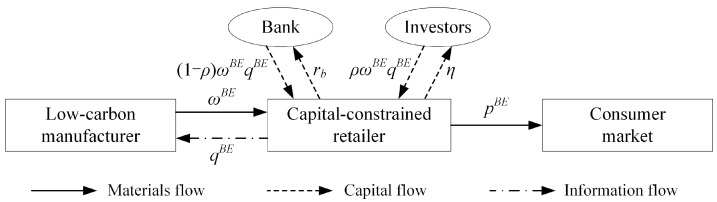
Hybrid financing strategy.

In this study, we focus on solving the issue of expensive financing costs for small and medium-sized retailers through a hybrid strategy that combines bank loan financing and equity financing, which will help to reduce financing costs and improve the efficiency of the low-carbon supply chain. Assuming that the retailer’s equity financing ratio is ρ, where ρ∈(0,1), then the proportion of bank loan financing is 1−ρ. The retailer allocates η of the investment income to institutional investors and repays the principal and interest to the bank in the amount of $\omega q\left(1+r_{b}\right)\left(1-\rho \right)$. Unlike those in Yang et al. [16], the equity financing ratio and equity dividend ratio are independent of each other, as shown in Figure 3.

The following equations can describe the profit functions of the manufacturer and the retailer:

(20) $$\pi_{m}^{BE}\left(\omega ,e\right)=\left(\omega -c_{m}\right)\left(\alpha -bp+\theta e\right)-\frac{1}{2}ke^{2},$$

(21) $$\pi_{r}^{BE}\left(p\right)=\left(1-\eta \right)\left(p-\omega \left(1+r_{b}\right)\left(1-\rho \right)\right))\left(\alpha -bp+\theta e\right).$$

In where superscript BE represents a hybrid financing strategy.

Under the hybrid financing strategy of bank loan and equity financing, we use a solution similar to that in Section 2.1.

By virtue of Equation (21), we can get that $\frac{\partial^{2}\pi_{r}^{BE}\left(p\right)}{\partial p^{2}}=-2b\left(1-\eta \right)\left(1+r_{b}\right)\left(1-\rho \right)<0$, so $\pi_{r}^{BE}\left(p\right)$ is a strictly concave function of *p*, then set the first derivative equal to zero from Equation (21), we obtain the optimal response function of the retailer:

(22) $$p=\frac{\alpha +\theta e+b\left(1+r_{b}\right)\left(1-\rho \right)\omega }{2b}.$$

Next, by virtue of Equation (20), we can get a Hessian Matrix of $\pi_{m}^{BE}\left(\omega ,e\right)$ as $H\left(\pi_{m}^{BE}\right)=\left[\begin{array}{cc} -b\left(1+r_{b}\right)\left(1-\rho \right) & \frac{\theta }{2}\\ \frac{\theta }{2} & -k \end{array}\right]$. Where $D_{1}\left(\pi_{m}^{BE}\right)=-b\left(1+r_{b}\right)\left(1-\rho \right)<0$, when $D_{2}\left(\pi_{m}^{BE}\right)=kb\left(1+r_{b}\right)\left(1-\rho \right)-\frac{\theta^{2}}{4}>0$, $\pi_{m}^{BE}\left(\omega ,e\right)$ is a strictly concave function of ω and *e*. Equating $\frac{\partial \pi_{m}^{BE}\left(\omega ,e\right)}{\partial \omega }=0$ and $\frac{\partial \pi_{m}^{BE}\left(\omega ,e\right)}{\partial e}=0$, we can obtain the optimal wholesale price and the level of carbon emissions reduction efforts:

(23) $${\omega^{BE}}^{*}=c_{m}+\frac{2k(\alpha -bc_{m}\left(1+r_{b}\right)\left(1-\rho \right))}{4kb\left(1+r_{b}\right)\left(1-\rho \right)-\theta^{2}},$$

(24) $${e^{BE}}^{*}=\frac{\theta (\alpha -bc_{m}\left(1+r_{b}\right)\left(1-\rho \right))}{4kb\left(1+r_{b}\right)\left(1-\rho \right)-\theta^{2}}.$$

Subsequently, put the values of ${\omega^{BE}}^{*}$ and ${e^{BE}}^{*}$ into Equations (1) and (22), we can get:

(25) $${p^{BE}}^{*}=c_{m}\left(1+r_{b}\right)\left(1-\rho \right)+\frac{3k\left(1+r_{b}\right)\left(1-\rho \right)\left(\alpha -bc_{m}\left(1+r_{b}\right)\left(1-\rho \right)\right)}{4kb\left(1+r_{b}\right)\left(1-\rho \right)-\theta^{2}},$$

(26) $${q^{BE}}^{*}=\frac{kb\left(1+r_{b}\right)\left(1-\rho \right)\left(\alpha -bc_{m}\left(1+r_{b}\right)\left(1-\rho \right)\right)}{4kb\left(1+r_{b}\right)\left(1-\rho \right)-\theta^{2}}.$$

Finally, we substitute Equations (23)–(26) into Equations (20) and (21), and obtain the maximum profits under the mixed financing mode of bank loan and equity financing:

(27) $${\pi_{m}^{BE}}^{*}=\frac{k{\left(\alpha -bc_{m}\left(1+r_{b}\right)\left(1-\rho \right)\right)}^{2}}{2(4kb\left(1+r_{b}\right)\left(1-\rho \right)-\theta^{2})}.$$

(28) $${\pi_{r}^{BE}}^{*}=\frac{\left(1-\eta \right)k^{2}b{\left(1+r_{b}\right)}^{2}{\left(1-\rho \right)}^{2}{\left(\alpha -bc_{m}\left(1+r_{b}\right)\left(1-\rho \right)\right)}^{2}}{{\left(4kb\left(1+r_{b}\right)\left(1-\rho \right)-\theta^{2}\right)}^{2}}.$$

**Property** **3.** *The impact of the equity financing ratio is given in the following results:*

$$\frac{\partial {e^{BE}}^{*}}{\partial \rho }>0,\frac{\partial {q^{BE}}^{*}}{\partial \rho }>0,\frac{\partial {\pi_{m}^{BE}}^{*}}{\partial \rho }>0,\frac{\partial {\pi_{r}^{BE}}^{*}}{\partial \rho }>0.$$

*The proof is given in Appendix A.3.*

Property 3 indicates that, in the low-carbon supply chain, when the retailer adopts the hybrid financing strategy, as the equity financing ratio (ρ) increases, the level of the manufacturer’s carbon emissions reduction efforts (${e^{BE}}^{*}$), the retailer’s order quantity (${q^{BE}}^{*}$), the manufacturer’s profit (${\pi_{m}^{BE}}^{*}$), and the retailer’s profit (${\pi_{r}^{BE}}^{*}$) increase accordingly.

In fact, a relatively high equity financing ratio means a low financing cost for the retailer and it borrows less from the bank and pays less interest to the bank, which reduces financial pressure on the retailer to repay the debt and interest due and enhances the competitiveness of the retailer. The investment institution will encourage the retailer to actively explore the market and increase the order volume; therefore, the retailer’s profit increases accordingly. The signal of a good market prospect encourages the manufacturer to make more carbon emissions reduction efforts. The manufacturer will raise the wholesale price of low-carbon products, which increases its profit, in order to ensure the maximization of profits and make up for the increase in the cost of carbon emissions reduction efforts brought about by market development.

**Property** **4.** *Now we discuss the impact of customers’ low-carbon preference in the three different financing strategies, we have the following results:*

$$\frac{\partial {e^{i}}^{*}}{\partial \theta }>0,\frac{\partial {q^{i}}^{*}}{\partial \theta }>0,\frac{\partial {\pi_{m}^{i}}^{*}}{\partial \theta }>0,\frac{\partial {\pi_{r}^{i}}^{*}}{\partial \theta }>0,inwhich\;\;i=\left\{B,E,BE\right\}.$$

*The proof is given in Appendix A.4.*

Property 4 shows that, regardless of financing strategy, as the coefficient of consumers’ low-carbon preference θ increases, the level of the manufacturer’s carbon emissions reduction efforts (${e^{i}}^{*}$), the retailer’s order quantity (${q^{i}}^{*}$), the manufacturer’s profit (${\pi_{m}^{i}}^{*}$), and the retailer’s profit (${\pi_{r}^{i}}^{*}$) increase.

The greater the consumer green preference coefficient, the better the market response to low-carbon products, which encourages the manufacturer to take more carbon emissions reduction efforts, thereby forming a benign low-carbon circular economy. In fact, the increase in demand for low-carbon products that is caused by consumers’ low-carbon preference is the fundamental driving force for the manufacturer to reduce carbon emissions, and the manufacturer’s investment in carbon emissions reduction (technical innovation, equipment and environmental improvements, etc.) creates tremendous economic and environmental benefits. The results of this study have verified the conclusions of references [3,36] that consumers’ low-carbon preference expands market demands. However, there is a difference: in this study, we took the impact of equity financing into consideration.

## 5. Selection of Financing Strategies

This section compares the above optimal results under the three financing strategies, namely, bank loan financing, equity financing, and hybrid financing. Through comparative analyses, we select the optimal strategy from the profits perspectives of the manufacturer and the retailer, and highlight the impact of the interest rate, the equity financing ratio and the equity dividend ratio.

### 5.1. Comparison of ω and E

**Proposition** **1.** *Under the three financing strategies, when the bank loan financing interest rate and equity financing ratio meet certain conditions, the optimal wholesale price and carbon emission reduction efforts of the manufacturer are in the following order:*

*1.*  *When $0\leq \rho \leq \frac{r_{b}}{1+r_{b}}$, then ${\omega^{B}}^{*}<{\omega^{BE}}^{*}\leq {\omega^{E}}^{*}$, ${e^{B}}^{*}<{e^{BE}}^{*}\leq {e^{E}}^{*}$;*
*2.*  *When $\frac{r_{b}}{1+r_{b}}<\rho \leq 1$, then ${\omega^{B}}^{*}<{\omega^{E}}^{*}<{\omega^{BE}}^{*}$, ${e^{B}}^{*}<{e^{E}}^{*}<{e^{BE}}^{*}$.*

*The proof is given in Appendix A.5.*

Proposition 1 indicates that, when the equity financing ratio is relatively low, the wholesale price is the highest in the equity financing strategy, followed by that in the hybrid financing strategy, and the lowest in the bank credit financing strategy. Whereas when the equity financing ratio is sufficiently high, the wholesale price is the highest in the mixed financing strategy, followed by that in the equity financing strategy, and is still the lowest in the bank credit financing strategy. The carbon emission reduction efforts also follow the same changing trend. This can be explained by the fact that the lower wholesale price is bad for both the manufacturer and their efforts to reduce carbon emission.

### 5.2. Comparison of q

**Proposition** **2.** *Under the three financing strategies, when the bank credit interest rate and equity financing ratio meet certain conditions, the optimal order quantity of the retailer is in the following order:*

*1.*  *When $0\leq \rho \leq \frac{r_{b}}{1+r_{b}}$, then ${q^{B}}^{*}<{q^{BE}}^{*}\leq {q^{E}}^{*}$;*
*2.*  *When $\frac{r_{b}}{1+r_{b}}<\rho \leq 1$, then ${q^{B}}^{*}<{q^{E}}^{*}<{q^{BE}}^{*}$.*

*The proof is given in Appendix A.6.*

Proposition 2 indicates that, when the equity financing ratio is relatively low, the actual demand of the market (the ordered quantity) is the highest in the equity financing strategy, followed by that in the hybrid financing strategy, and the lowest in the bank loan financing strategy. Whereas, when the equity financing ratio is sufficiently high, the ordered quantity is the highest in the hybrid financing strategy, followed by that in the equity financing strategy, and it is still the lowest in the bank loan financing strategy. From Proposition 2, we can see that the bank loan financing cannot stimulate the retailers to increase the ordered quantity, and the effect of expanding market demand is not significant.

### 5.3. Comparison of Profits

**Proposition** **3.** *Under the three financing strategies, when the bank loan interest rate and equity financing ratio meet certain conditions, the profit of the manufacturer is in the following order:*

*1.*  *When $0\leq \rho \leq \frac{r_{b}}{1+r_{b}}$, then ${\pi_{m}^{B}}^{*}<{\pi_{m}^{BE}}^{*}\leq {\pi_{m}^{E}}^{*}$;*
*2.*  *When $\frac{r_{b}}{1+r_{b}}<\rho \leq 1$, then ${\pi_{m}^{B}}^{*}<{\pi_{m}^{E}}^{*}<{\pi_{m}^{BE}}^{*}$.*

*The proof is given in Appendix A.7.*

Proposition 3 indicates that, no matter how the parameters ρ and $r_{b}$ change, under the bank loan financing, the profit of the manufacturer is still the lowest, we can see that, when the equity financing ratio is relatively low, the profit of the manufacturer of equity financing is higher than that of the hybrid financing, whereas, when the equity financing ratio is sufficiently high, the condition is just the opposite. This is enough to show that the manufacturer is not willing to choose the bank loan financing, preferring the equity financing or the hybrid financing. Equity financing can broaden the financing channel for the capital-constrained retailer, improve market competitiveness, relieve financial stress of debt repayment, and it is beneficial to the retailer. The well-funded retailer can expand the market aggressively, increase the order quantity. In this way, in order to make up for the increase of the carbon emission reduction cost brought by market development, the manufacturer will raise the wholesale price accordingly, and the increase of wholesale price and the order quantity will increase the profits of the manufacturer. This conclusion can provide a certain reference for the decision strategy of the manufacturer.

**Proposition** **4.** *Under the three financing strategies, the relationship of the retailer’s profit is dependent on three factors, namely, the bank loan interest rate, the equity financing ratio, and the equity dividend ratio.*

*There are two critical values:*

$$\eta^{*}=1-\frac{{\left(1+r_{b}\right)}^{2}{\left(\alpha -bc_{m}\left(1+r_{b}\right)\right)}^{2}{\left(4kb-\theta^{2}\right)}^{2}}{{\left(4kb\left(1+r_{b}\right)-\theta^{2}\right)}^{2}{\left(\alpha -bc_{m}\right)}^{2}}\;\;\;and$$

$$\eta^{**}=1-\frac{{\left(1+r_{b}\right)}^{2}{\left(\alpha -bc_{m}\left(1+r_{b}\right)\right)}^{2}{\left(4kbx-\theta^{2}\right)}^{2}}{{\left(4kb\left(1+r_{b}\right)-\theta^{2}\right)}^{2}{\left(\alpha -xbc_{m}\right)}^{2}x^{2}},$$

*where $x=\left(1+r_{b}\right)\left(1-\rho \right)$.*

*The profits of the retailer are in the following order:*

*1.*  *When $0\leq \rho \leq \frac{r_{b}}{1+r_{b}}$, $\eta^{*}\geq \eta^{**}$,*
  - *if $\eta \in (0,\eta^{**}]$, then ${\pi_{r}^{B}}^{*}\leq {\pi_{r}^{BE}}^{*}\leq {\pi_{r}^{E}}^{*}$;*
  - *if $\eta \in (\eta^{**},\eta^{*}]$, then ${\pi_{r}^{BE}}^{*}<{\pi_{r}^{B}}^{*}\leq {\pi_{r}^{E}}^{*}$;*
  - *if $\eta \in (\eta^{*},1]$, then ${\pi_{r}^{BE}}^{*}<{\pi_{r}^{E}}^{*}<{\pi_{r}^{B}}^{*}$.*
*2.*  *When $\frac{r_{b}}{1+r_{b}}<\rho \leq 1$, $\eta^{*}<\eta^{**}$, similarly,*
  - *if $\eta \in (0,\eta^{*}]$, then ${\pi_{r}^{B}}^{*}\leq {\pi_{r}^{E}}^{*}\leq {\pi_{r}^{BE}}^{*}$;*
  - *if $\eta \in (\eta^{*},\eta^{**}]$, then ${\pi_{r}^{E}}^{*}<{\pi_{r}^{B}}^{*}\leq {\pi_{r}^{BE}}^{*}$;*
  - *if $\eta \in (\eta^{**},1]$, then ${\pi_{r}^{E}}^{*}<{\pi_{r}^{BE}}^{*}<{\pi_{r}^{B}}^{*}$.*

*The proof is given in Appendix A.8.*

Proposition 4 indicates that the financing preference of the capital-constrained retailer is determined by three operating factors, namely, bank loan interest rate, equity financing ratio, and equity dividend ratio. There are twe critical values, $\eta^{*}$ and $\eta^{**}$. When the equity financing ratio is relatively low, i.e., $0\leq \rho \leq \frac{r_{b}}{1+r_{b}}$, then $\eta^{*}>\eta^{**}$. If $\eta \in (0,\eta^{**}]$, the retailer’s financing preference is in the following order: the optimal strategy is equity financing, then hybrid financing, and the last is bank loan financing; if $\eta \in (\eta^{**},\eta^{*}]$, the optimal strategy is still equity financing, then bank loan financing, the last is hybrid financing; if $\eta \in (\eta^{*},1)$, the retailer’s financing preference order has changed, the optimal strategy is bank loan financing, then equity financing, and the last is hybrid financing. Whereas, when the equity financing ratio is sufficiently high, i.e., $\frac{r_{b}}{1+r_{b}}<\rho \leq 1$, and $\eta^{*}<\eta^{**}$. If $\eta \in (0,\eta^{*}]$, the retailer’s financing preference is in the following order: the optimal strategy is hybrid financing, then equity financing, and the last is bank loan financing; if $\eta \in (\eta^{*},\eta^{**}]$, the optimal strategy is still hybrid financing, then bank loan financing, and the last is equity financing; if $\eta \in (\eta^{**},1)$, the optimal strategy is bank loan financing, the following is hybrid financing, and the last is equity financing. According to the relationship among bank loan interest rate, equity financing ratio, and equity dividend ratio, the optimal financing strategy is selected to provide some management insights for the capital-constrained retailer.

## 6. Numerical Analysis

**Figure 4:**
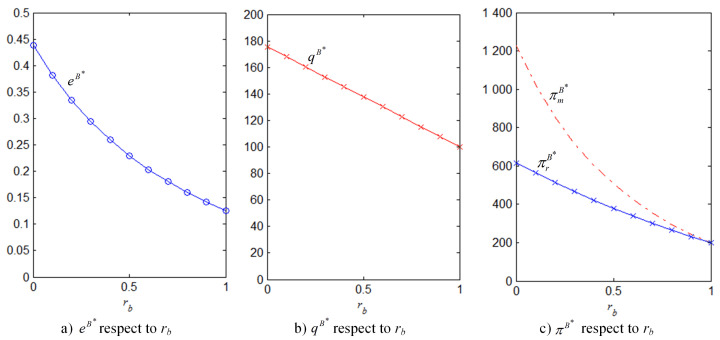
${e^{B}}^{*}$, ${q^{B}}^{*}$, ${\pi_{m}^{B}}^{*}$, ${\pi_{r}^{B}}^{*}$ with respect to $r_{b}$ (θ=5).

**Figure 5:**
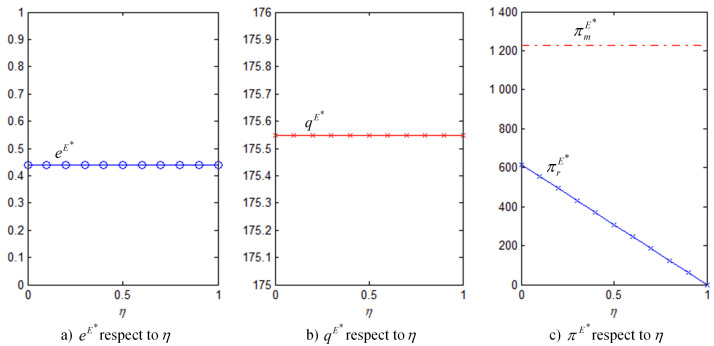
${e^{E}}^{*}$, ${q^{E}}^{*}$, ${\pi_{m}^{E}}^{*}$, ${\pi_{r}^{E}}^{*}$ with respect to η (θ=5, ρ=0.3).

**Figure 6:**
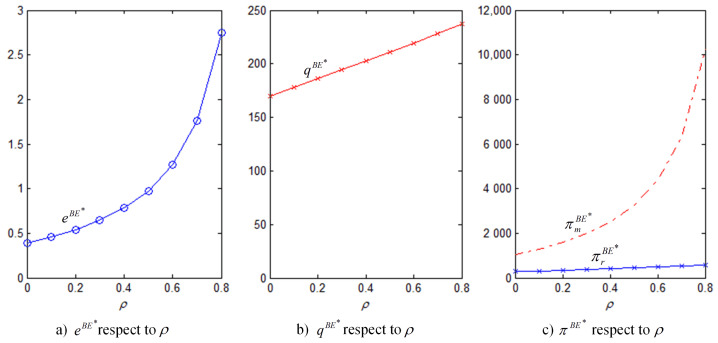
${e^{BE}}^{*}$, ${q^{BE}}^{*}$, ${\pi_{m}^{BE}}^{*}$, ${\pi_{r}^{BE}}^{*}$ with respect to ρ (θ=5, $r_{b}=0.1$, η=0.5).

**Figure 7:**
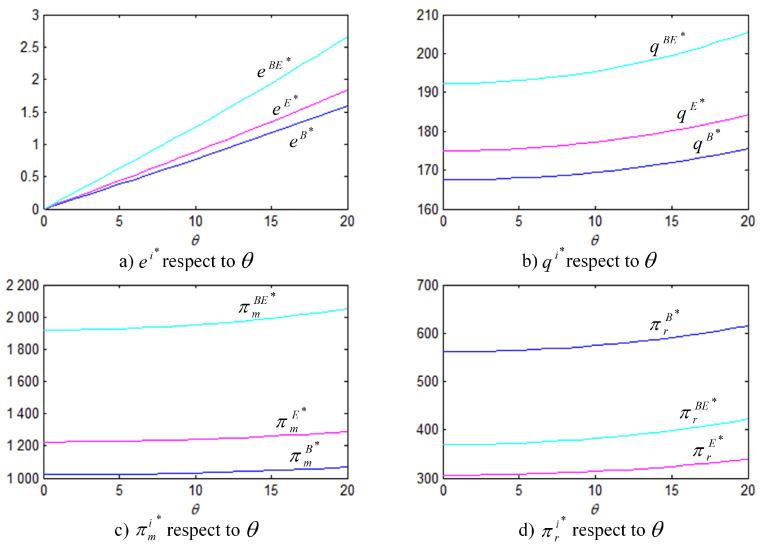
${e^{i}}^{*}$, ${q^{i}}^{*}$, ${\pi_{m}^{i}}^{*}$, ${\pi_{r}^{i}}^{*}$ with respect to θ ($r_{b}=0.1$, ρ=0.3, η=0.5, i=B,E,BE).

**Figure 8:**
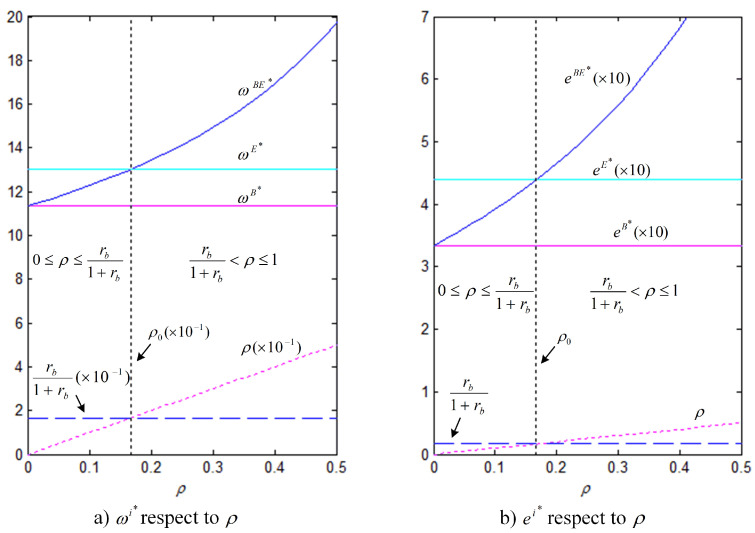
Comparison of the optimal ω and *e* under three financing strategies (θ=5, $r_{b}=0.2$).

**Figure 9:**
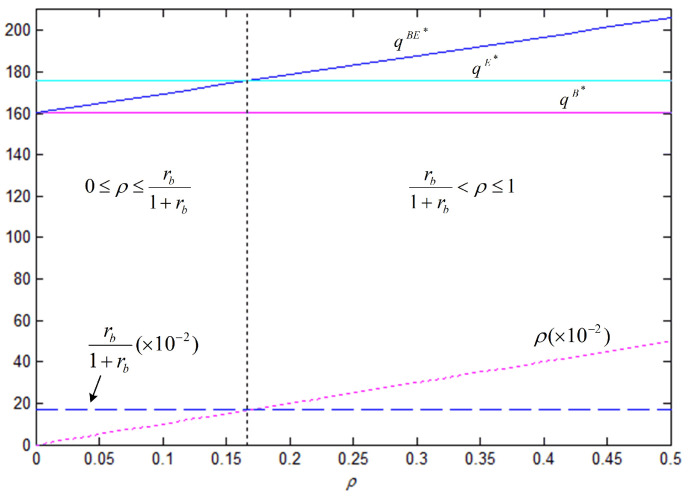
Comparison of the optimal *q* under three financing strategies (θ=5, $r_{b}=0.2$).

**Figure 10:**
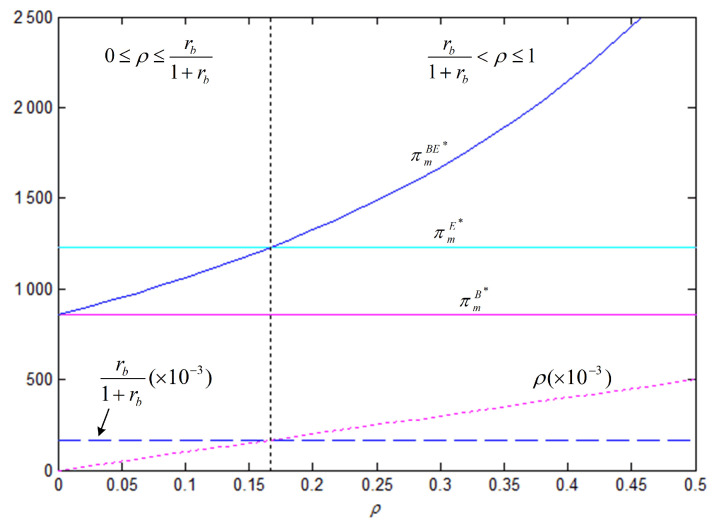
Comparison of the optimal $\pi_{m}$ under three financing strategies (θ=5, $r_{b}=0.2$).

**Figure 11:**
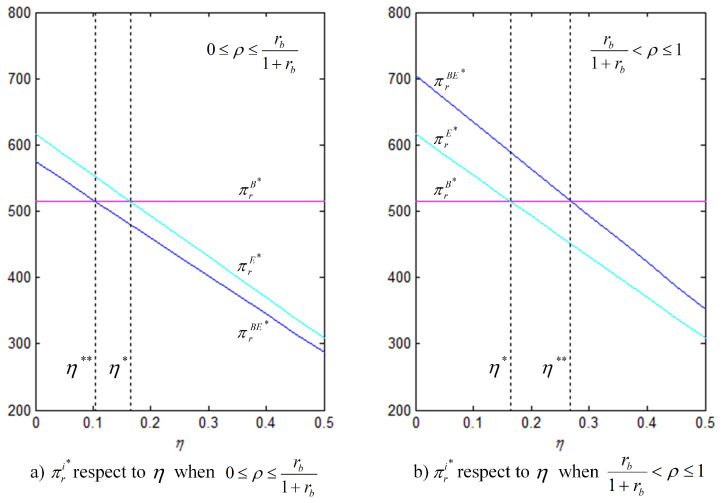
Comparison of the optimal $\pi_{r}$ under three financing strategies (θ=5, $r_{b}=0.2$, (**a**) ρ=0.1, (**b**) ρ=0.3).

In order to better illustrate the above properties and propositions further, we will verify them through numerical analysis. The main parameters are as follows: α=1000, b=50, $c_{m}=6$, k=40. This paper mainly analyzes the influences of the bank loan financing interest rate, the equity financing ratio, the equity dividend ratio and consumers’ low-carbon preference on decision-making variables and the order quantity, as well as the influences on the profits of low-carbon supply chain member enterprises. This conclusion can provide a certain reference for the decision financing strategy of the manufacturer and the retailer. The results are verified by numerical examples, and the details are shown in Figure 4, Figure 5, Figure 6, Figure 7, Figure 8, Figure 9, Figure 10 and Figure 11.

It can be observed from Figure 4 that the effects of bank loan financing interest rate on the optimal results of equilibrium under bank loan financing. Figure 4a demonstrates that the level of carbon emissions reduction efforts decreases with the interest rate. All of the other results of equilibrium have the same change, they all decrease with the interest rate in Figure 4b,c. Moreover, consistent with Property 1, the increase of the interest rate has negative effects on both the manufacturer and retailer’s profits and, thus, both of them are reluctant to choose bank loan financing.

Figure 5 shows that only the retailer’s profit is affected by the equity dividend ratio: the higher the equity dividend ratio, the less is the retailer’s residual income. The level of carbon emissions reduction efforts, the market ordering quantity, and the manufacturer’s profit do not change and they are consistent with those in the absence of financial constraints, verifying Property 2.

Figure 6 illustrates the effect of the equity financing ratio in the hybrid financing strategy on carbon emissions reduction efforts, order quantity, and corporate profit. The results show that the equity financing ratio has a positive impact on the manufacturer’s carbon emissions reduction efforts and profits, and the effect was significant. Therefore, the manufacturer may be more in favor of the hybrid financing strategy that combines equity financing and bank loan financing. However, the effect of the equity financing ratio on the retailer is not profound because the increase in market demand is not as fast as that in the wholesale price, i.e., the retailer bears a higher purchase cost.

Figure 7 shows that, regardless of financing strategy, the greater the coefficient of consumers’ low-carbon preference, the higher are the level of carbon emissions reduction efforts, the market order quantity, and the profits of the manufacturer and the retailer and that the emission reduction under hybrid financing is the most effective strategy. Figure 7c shows that the manufacturer always prefers the hybrid financing strategy, while the retailer, being affected by the equity dividend ratio, does not always opt for the hybrid financing strategy.

Figure 8 and Figure 9 show that, under the three financing strategies, the manufacturer’s wholesale price and level of carbon emissions reduction efforts and the retailer’s order quantity are jointly determined by three business factors, i.e., the interest rate of bank loan, the equity financing ratio, and the coefficient of consumers’ low-carbon preference. In Property 4, we analyzed the influence of the coefficient of consumers’ low-carbon preference. The results of the analysis show that there is a critical point ($\rho_{0}=\frac{r_{b}}{1+r_{b}}$) in the relationship between the equity financing ratio and the interest rate of bank loan financing. At low equity financing ratios, i.e., when $\rho <\rho_{0}$, we have ${\omega^{E}}^{*}>{\omega^{BE}}^{*}$, ${e^{E}}^{*}>{e^{BE}}^{*}$, and ${q^{E}}^{*}>{q^{BE}}^{*}$; as the equity financing ratio increases, when $\rho >\rho_{0}$, the opposite situation occurs. However, no matter how the equity financing ratio and the interest rate of bank loan financing change, the level of carbon emissions reduction efforts, the wholesale price, and the order quantity are always the smallest under the bank loan financing strategy.

Figure 10 shows that, when the retailer chooses the bank loan financing strategy, the manufacturer’s profit is always lower than that under the other two financing strategies; therefore, the manufacturer does not want the retailer to choose the bank loan financing strategy; when the retailer chooses the equity financing strategy, the manufacturer’s profit is the same as that in the case of no capital constraints, which is higher than that when bank loan financing is chosen. When the retailer chooses the hybrid financing strategy, the retailer’s marginal financing cost per unit of borrowed funds is $\left(1+r_{b}\right)\left(1-\rho \right)$, which means that a high equity financing ratio corresponds to a low financing cost. When the equity financing ratio is 0, the manufacturer’s profit remains the same as that when the bank loan financing strategy is chosen. As the equity financing ratio increases, the retailer, who has obtained more financial support, will increase the order quantity, allowing for the manufacturer to generate more revenue accordingly. Similar to the analysis results that are shown in Figure 8 and Figure 9, there also exists a critical point ($\rho_{0}=\frac{r_{b}}{1+r_{b}}$) in the equity financing ratio. At low equity financing ratios, i.e., when $\rho <\rho_{0}$, ${\pi_{m}^{E}}^{*}>{\pi_{m}^{BE}}^{*}$, which indicates that the manufacturer is most in favor of the retailer choosing equity financing; otherwise, when $\rho \geq \rho_{0}$, ${\pi_{m}^{E}}^{*}\leq {\pi_{m}^{BE}}^{*}$, indicating that the manufacturer is most in favor of the retailer choosing the hybrid financing strategy.

Figure 11 shows that, for the retailer, the equity dividend ratio and equity financing ratio jointly determine the choice of financing strategy. At low equity financing ratios, i.e., $\rho <\rho_{0}$ (in which, $\rho_{0}=\frac{r_{b}}{1+r_{b}}$), because, under the hybrid financing strategy, the retailer’s profit is always lower than that under equity financing and the retailer will not choose the hybrid financing strategy. At this point, there is a critical value $\eta^{*}$, and when $\eta \in (0,\eta^{*})$, the retailer prefers equity financing; otherwise, it prefers bank loan financing. As the equity financing ratio increases, when $\rho \geq \rho_{0}$, because the retailer’s profit under the hybrid financing strategy is higher than that under equity financing, the retailer’s financing preference will change. Similarly, there also exists a critical value $\eta^{**}$, and, when $\eta \in (0,\eta^{**})$, the retailer prefers hybrid financing; otherwise, it is inclined to choose bank loan financing.

## 7. Conclusions

In this study, we examined the financing strategy of the capital-constrained supply chain while taking consumers’ low-carbon preference into account. The manufacturer invests in carbon emissions reduction technologies and produces low-carbon products, and the capital-constrained retailer orders and sells these low-carbon products. To determine the impact of carbon emissions reduction on supply chain operations and financing decisions and to solve retailers’ financial constraints, we analyzed three financing strategies, i.e., bank loan financing, equity financing, and hybrid financing, which is different from the research on bank loans, trade credit, and mixed financing designed by Li and Yang [31,36]. We constructed game models for each strategy and inversely solved the equilibrium results for the wholesale price, the level of carbon emissions reduction efforts, the order quantity, and participant’s profits under the three financing strategies.

The above equilibrium results have several interesting management implications. First, the analysis of the influence of consumers’ low-carbon preference, the interest rate of bank loan, the equity financing ratio, and the equity dividend ratio on the equilibrium results indicates that the increase in the consumers’ low-carbon preference has a positive effect on the equilibrium outcomes, which is similar to the prior studies of [4,5,17,18,19,20,21,22,23], while an increase in the interest rate of bank loan financing has the opposite effect; similarly, the equity financing ratio also has a positive effect on them; the equity dividend ratio only has an effect on the retailer’s profit but no effect on other equilibrium results. Different from the prior studies above, the influence of consumers’ low-carbon preferences in the capital-constrained low-carbon supply chain along with bank loan interest rate, equity financing ratio, and equity dividend ratio on the equilibrium results is considered in the analysis, which is the highlight of this work. In addition, equity financing ratio and equity dividend ratio are independent variables in this research, which is different from the researches of [9,16,31,36]. Second, the comparison of the wholesale prices as well as the levels of carbon emissions reduction efforts and order quantities under the three financing strategies indicates that, at high equity financing ratios, the optimal value of the above decision variables appears under hybrid financing; otherwise, it appears under equity financing. When the equity financing ratio is low, the manufacturer always prefers equity financing. The retailer prefers equity financing when the equity dividend ratio is low; otherwise, it prefers bank loan financing. When the equity financing ratio is high, the manufacturer prefers hybrid financing. The retailer prefers hybrid financing when the equity dividend ratio is low, otherwise, it prefers bank loan financing. Regardless of the equity financing ratio and the equity dividend ratio, the manufacturer always does not want the retailer to choose bank loan financing.

This study has some limitations. First, we only analyzed external financing strategies of a supply chain and ignored the internal financing strategies, such as trade credits. Second, in this study, we only considered one manufacturer and one capital-constrained retailer, and the effect of the competition between multiple retailers on the equilibrium results needs to be examined further. Finally, we assumed that the demand function is fixed. However, in reality, various random factors may affect the demand function. Therefore, incorporating randomness into the demand function is another direction that is worth studying.

## Figures and Tables

**Table 1:** Notations definition.

Parameters	Model Parameters
*q*	The actual demand of the market
α	The potential size of the total market demand
*b*	The sensitivity of the consumers to the retail price
θ	The coefficient of the consumers’ low-carbon preference
*k*	The carbon emission cost parameter, *k* is a constant
$c_{m}$	The manufacturer’s unit production cost
$r_{b}$	The interest rate of bank loan financing
η	Equity dividend ratio
ρ	Equity financing ratio
$\pi_{j}^{i}$	The profits of low-carbon supply chain member enterprises ${}^{a}$
	**Decision variables**
ω	Unit wholesale price of the products
*e*	The level of carbon emissions reduction efforts
*p*	The retail price of the products

^a^*i* = {*B*, *E*, *BE*}, *j* = {*m*, *r*} represent the profits of the manufacturer and the retailer under bank loan financing, equity financing and hybrid financing respectively.

**Figure A1:**
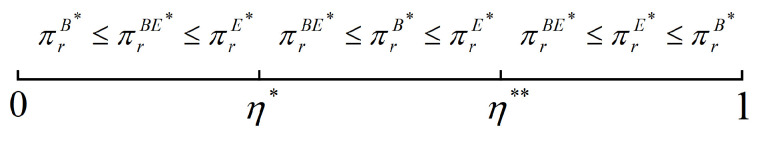
The relationship among ${\pi_{r}^{B}}^{*}$, ${\pi_{r}^{E}}^{*}$ and ${\pi_{r}^{BE}}^{*}$ when x≥1.

**Figure A2:**
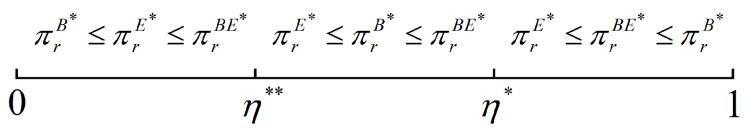
The relationship among ${\pi_{r}^{B}}^{*}$, ${\pi_{r}^{E}}^{*}$ and ${\pi_{r}^{BE}}^{*}$ when x<1.

## Data Availability

Not applicable.

## Appendix A. Proof of Properties

### Appendix A.1. Proof of Property 1

By virtue of Equations (6) and (8)–(10), we can get that

(A1) $$\frac{\partial {e^{B}}^{*}}{\partial r_{b}}=-\frac{\theta (bc_{m}\left(4kb\left(1+r_{b}\right)-\theta^{2}\right)+4kb\left(\alpha -bc_{m}\left(1+r_{b}\right)\right))}{{\left(4kb\left(1+r_{b}\right)-\theta^{2}\right)}^{2}}<0,$$

(A2) $$\frac{\partial {q^{B}}^{*}}{\partial r_{b}}=-\frac{kb(bc_{m}\left(4kb\left(1+r_{b}\right)-\theta^{2}\right)+\theta^{2}\left(\alpha -bc_{m}\left(1+r_{b}\right)\right))}{{\left(4kb\left(1+r_{b}\right)-\theta^{2}\right)}^{2}}<0.$$

Let $\Phi_{1}=\alpha -bc_{m}\left(1+r_{b}\right)>0$ and $\Phi_{2}=\frac{\alpha -bc_{m}\left(1+r_{b}\right)}{4kb\left(1+r_{b}\right)-\theta^{2}}>0$, then ${\pi_{m}^{B}}^{*}=\frac{k}{2}\Phi_{1}\Phi_{2}$, and the first-order partial derivative of ${\pi_{m}^{B}}^{*}$ with respect to $r_{b}$ is

(A3) $$\frac{\partial {\pi_{m}^{B}}^{*}}{\partial r_{b}}=\frac{k}{2}\left(\frac{\partial \Phi_{1}}{\partial r_{b}}\Phi_{2}+\Phi_{1}\frac{\partial \Phi_{2}}{\partial r_{b}}\right).$$

For $\frac{\partial \Phi_{1}}{\partial r_{b}}=-bc_{m}<0$ and $\frac{\partial \Phi_{2}}{\partial r_{b}}=-\frac{bc_{m}\left(4kb\left(1+r_{b}\right)-\theta^{2}\right)+4kb\left(\alpha -bc_{m}\left(1+r_{b}\right)\right)}{{\left(4kb\left(1+r_{b}\right)-\theta^{2}\right)}^{2}}<0$,

(A4) $$\frac{\partial {\pi_{m}^{B}}^{*}}{\partial r_{b}}<0.$$

Similarly, by the same token,

(A5) $$\frac{\partial {\pi_{r}^{B}}^{*}}{\partial r_{b}}<0.$$

### Appendix A.2. Proof of Property 2

From Equations (15) and (17)–(19), we can obtain that

$\frac{\partial {e^{E}}^{*}}{\partial \eta }=0$, $\frac{\partial {q^{E}}^{*}}{\partial \eta }=0$, $\frac{\partial {\pi_{m}^{E}}^{*}}{\partial \eta }=0$, $\frac{\partial {\pi_{r}^{E}}^{*}}{\partial \eta }=-\frac{k^{2}b{\left(\alpha -bc_{m}\right)}^{2}}{{\left(4kb-\theta^{2}\right)}^{2}}<0$.

### Appendix A.3. Proof of Property 3

From Equations (24) and (26)–(28), we can obtain that

(A6) $$\frac{\partial {e^{BE}}^{*}}{\partial \rho }=\frac{\theta \left(1+r_{b}\right)\left(bc_{m}\left(4kb\left(1+r_{b}\right)\left(1-\rho \right)-\theta^{2}\right)+4kb\left(\alpha -bc_{m}\left(1+r_{b}\right)\left(1-\rho \right)\right)\right)}{{\left(4kb\left(1+r_{b}\right)\left(1-\rho \right)-\theta^{2}\right)}^{2}}>0,$$

(A7) $$\frac{\partial {q^{BE}}^{*}}{\partial \rho }=\frac{kb\left(1+r_{b}\right)\left(bc_{m}\left(1+r_{b}\right)\left(1-\rho \right)\left(4kb\left(1+r_{b}\right)\left(1-\rho \right)-\theta^{2}\right)+\theta^{2}\left(\alpha -bc_{m}\left(1+r_{b}\right)\left(1-\rho \right)\right)\right)}{{\left(4kb\left(1+r_{b}\right)\left(1-\rho \right)-\theta^{2}\right)}^{2}}>0.$$

Let $\Phi_{3}=\alpha -bc_{m}\left(1+r_{b}\right)\left(1-\rho \right)>0$ and $\Phi_{4}=\frac{\alpha -bc_{m}\left(1+r_{b}\right)\left(1-\rho \right)}{4kb\left(1+r_{b}\right)\left(1-\rho \right)-\theta^{2}}>0$, then ${\pi_{m}^{BE}}^{*}=\frac{k}{2}\Phi_{3}\Phi_{4}$, and the first-order partial derivative of ${\pi_{m}^{BE}}^{*}$ with respect to ρ is

(A8) $$\frac{\partial {\pi_{m}^{BE}}^{*}}{\partial \rho }=\frac{k}{2}\left(\frac{\partial \Phi_{3}}{\partial \rho }\Phi_{4}+\Phi_{3}\frac{\partial \Phi_{4}}{\partial \rho }\right).$$

In the same way with Appendix A.1, it can be calculated that $\frac{\partial \Phi_{3}}{\partial \rho }>0$, $\frac{\partial \Phi_{4}}{\partial \rho }>0$, then

(A9) $$\frac{\partial {\pi_{m}^{BE}}^{*}}{\partial \rho }>0.$$

In addition, we can see that $\frac{\partial {\pi_{r}^{BE}}^{*}}{\partial \rho }=2\left(1-\eta \right)k^{2}b{\left(1+r_{b}\right)}^{2}{q^{BE}}^{*}\frac{\partial {q_{m}^{BE}}^{*}}{\partial \rho }$, so

(A10) $$\frac{\partial {\pi_{r}^{BE}}^{*}}{\partial \rho }>0.$$

### Appendix A.4. Proof of Property 4

When i=B, from Equations (6) and (8)–(10), we can obtain that:

(A11) $$\frac{\partial {e^{B}}^{*}}{\partial \theta }=\frac{\left(4kb\left(1+r_{b}\right)+\theta^{2}\right)\left(\alpha -bc_{m}\left(1+r_{b}\right)\right)}{{\left(4kb\left(1+r_{b}\right)-\theta^{2}\right)}^{2}}>0,$$

(A12) $$\frac{\partial {q^{B}}^{*}}{\partial \theta }=\frac{2kb\theta \left(1+r_{b}\right)\left(\alpha -bc_{m}\left(1+r_{b}\right)\right)}{{\left(4kb\left(1+r_{b}\right)-\theta^{2}\right)}^{2}}>0,$$

(A13) $$\frac{\partial {\pi_{m}^{B}}^{*}}{\partial \theta }=\frac{k\theta {\left(\alpha -bc_{m}\left(1+r_{b}\right)\right)}^{2}}{{\left(4kb\left(1+r_{b}\right)-\theta^{2}\right)}^{2}}>0,$$

(A14) $$\frac{\partial {\pi_{r}^{B}}^{*}}{\partial \theta }=\frac{4k^{2}b\theta {\left(1+r_{b}\right)}^{2}{\left(\alpha -bc_{m}\left(1+r_{b}\right)\right)}^{2}}{{\left(4kb\left(1+r_{b}\right)-\theta^{2}\right)}^{3}}>0.$$

And in the same way, when i=E,BE, we can get the similar conclusion.

### Appendix A.5. Proof of Proposition 1

When ρ=0, we can see that ${\omega^{B}}^{*}={\omega^{BE}}^{*}$, by virtue of $\frac{\partial {\omega^{BE}}^{*}}{\partial \rho }>0$, ${\omega^{B}}^{*}<{\omega^{BE}}^{*}$. While when $r_{b}=0$, we can see that ${\omega^{B}}^{*}={\omega^{E}}^{*}$, similarly, for $\frac{\partial {\omega^{B}}^{*}}{\partial r_{b}}<0$, ${\omega^{B}}^{*}<{\omega^{E}}^{*}$.

Let $x=\left(1+r_{b}\right)\left(1-\rho \right)$, and ${\omega^{E}}^{*}-{\omega^{BE}}^{*}=\frac{2k\left(4\alpha kb-bc_{m}\theta^{2}\right)\left(x-1\right)}{\left(4kb-\theta^{2}\right)\left(4kbx-\theta^{2}\right)}$. From Assumptions 1 and 3, we can easily get $\alpha -bc_{m}>0$. To ensure the concave of function $\pi_{m}^{E}\left(\omega ,e\right)$, it can be seen from the Hessian Matrix that $4kb-\theta^{2}>0$, so $4\alpha kb-bc_{m}\theta^{2}>0$. If x≥1, i.e., $0\leq \rho \leq \frac{r_{b}}{1+r_{b}}$, we can see that ${\omega^{E}}^{*}\geq {\omega^{BE}}^{*}$, so ${\omega^{B}}^{*}<{\omega^{BE}}^{*}\leq {\omega^{E}}^{*}$, or else ${\omega^{E}}^{*}<{\omega^{BE}}^{*}$, ${\omega^{B}}^{*}<{\omega^{E}}^{*}<{\omega^{BE}}^{*}$. And in the same way, we can also get the conclusion that when x≥1, i.e. $0\leq \rho \leq \frac{r_{b}}{1+r_{b}}$, ${e^{B}}^{*}<{e^{BE}}^{*}\leq {e^{E}}^{*}$, or else ${e^{B}}^{*}<{e^{E}}^{*}\leq {e^{BE}}^{*}$.

### Appendix A.6. Proof of Proposition 2

When ρ=0, we can see that ${q^{B}}^{*}={q^{BE}}^{*}$, by virtue of $\frac{\partial {q^{BE}}^{*}}{\partial \rho }>0$, so ${q^{B}}^{*}<{q^{BE}}^{*}$, further, ${q^{E}}^{*}-{q^{BE}}^{*}=\frac{kb\left(\alpha -bc_{m}\right)\theta^{2}+bc_{m}\left(4kb-\theta^{2}\right)x)\left(x-1\right)}{4kb-\theta^{2}}$, referring to the proof of Proposition 1, we can get the similar conclusion.

### Appendix A.7. Proof of Proposition 3

From Equation (27), in which $x=\left(1+r_{b}\right)\left(1-\rho \right)$, so ${\pi_{m}^{BE}}^{*}\left(x\right)=\frac{k{\left(\alpha -bc_{m}x\right)}^{2}}{2(4kbx-\theta^{2})}$, so $\frac{\partial {\pi_{m}^{BE}}^{*}\left(x\right)}{\partial x}<0$. That is to say ${\pi_{m}^{BE}}^{*}\left(x\right)$ is a monotonically decreasing function of *x*. If x=1, we can get that ${\pi_{m}^{E}}^{*}={\pi_{m}^{BE}}^{*}$. Further more, when x≥1, similar to Proposition 1, it can be obtained that ${\pi_{m}^{B}}^{*}\leq {\pi_{m}^{BE}}^{*}<{\pi_{m}^{E}}^{*}$, otherwise, when x<1, we can get ${\pi_{m}^{B}}^{*}<{\pi_{m}^{E}}^{*}<{\pi_{m}^{BE}}^{*}$.

### Appendix A.8. Proof of Proposition 4

When η=0, the retailer doesn’t pay any dividends to equity investors, it is easy to get the conclusion that ${\pi_{r}^{B}}^{*}<{\pi_{r}^{E}}^{*}$. As the equity dividend ratio increases, the retailer’s profits will become smaller and smaller under the equity financing strategy. There is a critical value $\eta^{*}=1-\frac{{\left(1+r_{b}\right)}^{2}{\left(\alpha -bc_{m}\left(1+r_{b}\right)\right)}^{2}{\left(4kb-\theta^{2}\right)}^{2}}{{\left(4kb\left(1+r_{b}\right)-\theta^{2}\right)}^{2}{\left(\alpha -bc_{m}\right)}^{2}}$, when $\eta =\eta^{*}$, ${\pi_{r}^{B}}^{*}={\pi_{r}^{E}}^{*}$. Similarly, there is another critical value $\eta^{**}=1-\frac{{\left(1+r_{b}\right)}^{2}{\left(\alpha -bc_{m}\left(1+r_{b}\right)\right)}^{2}{\left(4kbx-\theta^{2}\right)}^{2}}{{\left(4kb\left(1+r_{b}\right)-\theta^{2}\right)}^{2}{\left(\alpha -xbc_{m}\right)}^{2}x^{2}}$, in which, $x=\left(1+r_{b}\right)\left(1-\rho \right)$. When $\eta =\eta^{**}$, ${\pi_{r}^{B}}^{*}={\pi_{r}^{BE}}^{*}$. It can be seen further that $\eta^{*}-\eta^{**}=\frac{{\left(1+r_{b}\right)}^{2}{\left(\alpha -bc_{m}\left(1+r_{b}\right)\right)}^{2}\left(\left(4kbx-\theta^{2}\right)\left(\alpha -bc_{m}\right)+x\left(\alpha -xbc_{m}\right)\left(4kb-\theta^{2}\right)\right)\left(\theta^{2}\left(\alpha -bc_{m}\right)+xbc_{m}\left(4kb-\theta^{2}\right)\right)\left(x-1\right)}{{\left(4kb\left(1+r_{b}\right)-\theta^{2}\right)}^{2}{\left(\alpha -xbc_{m}\right)}^{2}x^{2}{\left(\alpha -bc_{m}\right)}^{2}}$, from Assumptions 1 and 3, we can get $\alpha -bc_{m}>0$, $\alpha -xbc_{m}>0$. To ensure the concave of function $\pi_{m}^{i}\left(\omega ,e\right)$ (where i=B,E,BE), it can be seen from the Hessian Matrixes that $4kb\left(1+r_{b}\right)-\theta^{2}>0$, $4kb-\theta^{2}>0$, $4kbx-\theta^{2}>0$. The relationship between $\eta^{*}$ and $\eta^{**}$ depends on value of *x*. When x≥1, i.e., $0\leq \rho \leq \frac{r_{b}}{1+r_{b}}$, $\eta^{*}\geq \eta^{**}$, otherwise, we can get $\eta^{*}<\eta^{**}$. It can be seen further that

${\pi_{r}^{E}}^{*}-{\pi_{r}^{BE}}^{*}=\left(1-\eta \right)k^{2}b\left(\frac{\alpha -bc_{m}}{4kb-\theta^{2}}+\frac{x(\alpha -xbc_{m})}{4kbx-\theta^{2}}\right)\left(\frac{\alpha -bc_{m}}{4kb-\theta^{2}}-\frac{x(\alpha -xbc_{m})}{4kbx-\theta^{2}}\right)=\left(1-\eta \right)k^{2}b\left(\frac{\alpha -bc_{m}}{4kb-\theta^{2}}+\frac{x(\alpha -xbc_{m})}{4kbx-\theta^{2}}\right)\frac{\left(bc_{m}x\left(4kb-\theta^{2}\right)+\theta^{2}\left(\alpha -bc_{m}\right)\right)}{\left(4kb-\theta^{2}\right)\left(4kbx-\theta^{2}\right)}\left(x-1\right)$. In summary, similarly to Proposition 1, when x≥1, i.e., $0\leq \rho \leq \frac{r_{b}}{1+r_{b}}$, the relationship among ${\pi_{r}^{B}}^{*}$, ${\pi_{r}^{E}}^{*}$ and ${\pi_{r}^{BE}}^{*}$ is shown in Figure A1; whlie when x<1, i.e., $\frac{r_{b}}{1+r_{b}}\leq \rho \leq 1$, the relationship among ${\pi_{r}^{B}}^{*}$, ${\pi_{r}^{E}}^{*}$ and ${\pi_{r}^{BE}}^{*}$ is shown in Figure A2, which can verify Propositon 4.
